# Inpatient psychiatric care of COVID-19 infected patients in a Hungarian general hospital

**DOI:** 10.1192/j.eurpsy.2022.530

**Published:** 2022-09-01

**Authors:** G. Gazdag, Z. Grenda, R. Takács

**Affiliations:** Jahn Ferenc South-pest Hospital, Centre Of Psychiatry, Budapest, Hungary

**Keywords:** Covid-19, inpatient psychiatric care, outcome

## Abstract

**Introduction:**

during the study period (08/02/2021 – 11/05/2021) the Centre of Psychiatry in the Jahn Ferenc South-pest Hospital (CP-JFSH) was one of the two psychiatric wards in Budapest, specialized for the treatment of COVID-19 infected psychiatric patients.

**Objectives:**

the aim of the study was to survey the characteristics and evaluate the outcome of the COVID-19 infected psychiatric patients treated in the CP-JFSH.

**Methods:**

retrospective analysis of the files of COVID-19 infected psychiatric patients admitted to the CP-JFSH in a 3 month period. In addition to demographic data, diagnostic distribution, co-morbidities, date of infection, method of detection of the virus, presence of pneumonia, severity of infection, outcome, treatment, vaccination data were evaluated.

**Results:**

in the study period 124 COVID-19 infected psychiaric patients were admitted to the CP-JFSH. The gender distribution was aproximately equal, the mean age of the patients was 62.8+/-15.7 years. Majority of the patients suffered from major neurocognitive disorder followed by schizophrenia spectrum disorder. Most common co-morbidities were cardiovascular diseases and diabetes. Pneumonia was present in 41% of the patients. Majority of the patients were already infected at the time of admission, detected with the first PCR examination and haven’t been vaccinated yet. Thirty-one percent of the patients suffered from moderate to severe COVID-19 illness. COVID-19 specific therapy (favipiravir, remdesivir, fluvoxamin) was introduced in 57%. Mortality was 12% while the relaps rate 4%.

**Conclusions:**

comparing with inpatient mortality rate published in the literature, mortality rate was higher among psychiatric patients, underlining the need for special attention of this population.

**Disclosure:**

No significant relationships.

